# From protocol to practice: long-Term outcomes of single-Fraction stereotactic body radiotherapy for primary non-Small cell lung cancer

**DOI:** 10.1007/s00066-025-02462-4

**Published:** 2025-09-04

**Authors:** Kerem Tuna Tas, Philipp Lishewski, Fatima Frosan Sheikhzadeh, Edgar Smalec, Niklas Recknagel, Thomas Wündisch, Angelique Holland, Andreas Kirschbaum, Khaled Elsayad, Rita Engenhart-Cabillic, Klemens Zink, Hilke Vorwerk, Sebastian Adeberg, Ahmed Gawish

**Affiliations:** 1 Department of Radiotherapy and Radiation Oncology, Marburg University Hospital, Marburg, Germany; 2 Department of Radiotherapy and Radiation Oncology, Philipps-Universität Marburg, Marburg, Germany; 3 Marburg Ion-Beam Therapy Center (MIT), Department of Radiotherapy and Radiation Oncology, Marburg University Hospital, Marburg, Germany; 4 University Cancer Center (UCT) Frankfurt—Marburg, Marburg, Germany; 5 LOEWE Research Cluster for Advanced Medical Physics in Imaging and Therapy, (ADMIT), TH Mittel Hessen University of Applied Sciences, Giessen, Germany; 6 Department of Medicine, Pulmonary and Critical Care Medicine, University Medical Center Giessen and Marburg, Philipps-University Marburg, Marburg, Germany; 7 Department of Visceral, Thoracic and Vascular Surgery, University Hospital Gießen and Marburg (UKGM), Marburg, Germany

**Keywords:** NSCLC, SBRT, Stereotactic radiosurgery, Single-fraction, Lung cancer, Radiation oncology

## Abstract

**Background:**

Single-fraction stereotactic body radiotherapy (SBRT) is an effective treatment option for patients with non-small cell lung cancer (NSCLC) who are ineligible for surgery. This study investigates long-term clinical outcomes, prognostic factors, and toxicity associated with high-dose single-fraction SBRT.

**Materials and methods:**

We retrospectively analyzed 110 patients with 116 NSCLC lesions treated with single-fraction SBRT between 2000 and 2023. Histologic subtypes included adenocarcinoma, squamous cell carcinoma, large cell carcinoma, and CT-defined suspicious lesions without histological confirmation. Local control (LC), progression-free survival (PFS), and overall survival (OS) were assessed using Kaplan-Meier and Cox regression models. Toxicity was evaluated using CTCAE v4.0.

**Results:**

The most common dose was 30 Gy, prescribed in 76.7% of lesions. Among patients who received ≥ 30 Gy, LC at 2, 3, and 5 years was 78%, 74%, and 68%; PFS was 63%, 49%, and 37%; and OS was 84%, 83%, and 60%, respectively. LC and PFS were significantly higher in patients treated with ≥ 30 Gy (*p* < 0.05).

Acute pneumonitis occurred in 2 patients (1.8%), and 22 patients (20.0%) developed late-onset pneumonitis. Pneumonitis incidence was 26.8% in patients planned with 3D-CT, compared to 12.8% with DIBH or 4D-CT. No grade ≥ 3 toxicity was observed.

**Conclusion:**

High-dose (≥ 30 Gy) single-fraction SBRT provides excellent long-term tumor control with minimal toxicity with NSCLC. Advanced motion management techniques were associated with reduced pulmonary toxicity. A ≥ 30 Gy dose significantly improved LC, PFS, and OS. Higher Charlson Comorbidity Index (CCI) was associated with worse OS. These findings support the use of high-dose SF-SBRT in selected patients and highlight the need for individualized treatment planning. Prospective validation is warranted.

## Introduction

Stereotactic body radiation therapy (SBRT) is recognized as an effective treatment for patients with early-stage non-small cell lung cancer (NSCLC) who are not candidates for surgery, achieving local control (LC) rates exceeding 90% within two years [1–3]. These outcomes are comparable to those achieved with surgical treatments. Advances in radiation oncology techniques and extensive clinical experience have enabled the safe and accurate delivery of SBRT, resulting in a low incidence of severe acute pneumonitis, reported at 1.6–4.2% [4, 5]. While surgery offers benefits such as lymph node sampling and the identification of metastases that may not be detectable on imaging, some studies, including pooled analyses and extended single-arm SBRT cohorts, have provided comparative insights. These studies suggest that LC rates between the two modalities are similar [6–10].

SBRT is conventionally delivered in multiple fractions, typically ranging from 3 to 10 sessions. However, recent evidence suggests that a single-dose regimen may offer distinct advantages, including improved convenience for patients, reduced risk of positioning errors, decreased equipment and cost demands, and seamless integration with systemic therapies. Additionally, single high-dose radiation may exert indirect tumoricidal effects by disrupting tumor vasculature. Despite these advantages, the adoption of single-dose SBRT has been limited due to concerns about severe toxicity and insufficient long-term data on its efficacy, although reported rates of severe acute toxicity remain relatively low.

The COVID-19 pandemic has heightened interest in single-fraction SBRT, given its potential to reduce patient visits and resource use. Emerging evidence from prospective and retrospective studies, as well as ongoing randomized trials, has further underscored its relevance. Current guidelines from the European Society for Radiotherapy and Oncology (ESTRO) and the American Society for Radiation Oncology (ASTRO) support the exploration of single-fraction SBRT for select patients, particularly those with peripheral early-stage NSCLC [11].

Although existing studies demonstrate promising tolerability and high LC rates with single-fraction SBRT, data on long-term outcomes and specific patient or tumor characteristics to guide individualized treatment remain limited. This retrospective study aims to address these gaps by comparing two single-dose SBRT protocols (≥ 30 Gy and < 30 Gy) with a focus on long-term follow-up to evaluate oncological outcomes and toxicity.

The Charlson Comorbidity Index (CCI) is a widely used and validated scoring system that predicts the risk of mortality based on the presence and severity of comorbid conditions. It was originally developed in a cohort of 559 patients and demonstrated a stepwise increase in mortality with higher scores, which was further confirmed in a 10-year follow-up cohort.13 CCI enables standardized risk stratification in longitudinal studies and has been shown to correlate with overall survival in various cancer populations.

This study is among the first to evaluate the prognostic value of CCI in patients with NSCLC treated with single-fraction SBRT, making it a novel contribution to the literature. Understanding the relationship between comorbidity burden and survival is particularly important for guiding individualized treatment decisions in patients with high comorbidity. The findings may support more accurate risk assessment and help clarify the role of SBRT in patients who are not candidates for surgery.

## Material and methods

This retrospective study analyzed all patients diagnosed with NSCLC who received single-fraction SBRT at our institution between 2000 and 2023. Patient selection followed a standardized institutional protocol and involved a multidisciplinary tumor board including thoracic surgeons, radiation oncologists, medical oncologists, pulmonologists, radiologists, and pathologists.

A total of 110 patients with 116 pulmonary lesions were included. Of these, 86 lesions (74.1%) were histologically confirmed NSCLC, while 30 lesions (25.9%) were CT-defined, morphologically suspicious round nodules. These lesions were treated based on strong clinical and radiological evidence of malignancy, such as 18-fluorodeoxyglucose-positron emission tomography (FDG-PET/CT) uptake, documented lesion growth, and/or high-risk clinical profiles. In all cases, the morphological appearance on CT was highly consistent with NSCLC. Furthermore, treatment decisions were confirmed by a multidisciplinary tumor board, which assessed each case and concluded that an NSCLC diagnosis was highly likely. Therefore, despite the lack of histological confirmation, inclusion of these patients in the NSCLC cohort was considered justified.

The inclusion criteria for this study were as follows: (1) a performance status of ECOG (Eastern Cooperative Oncology Group) ≤ 2; (2) ineligibility for surgery due to advanced age, comorbidities, or refusal of invasive procedures; (3) absence of other active disease sites, including locoregional lymph nodes or distant metastases.

Pre-treatment evaluation included clinical examination, pulmonary function testing, a total body CT scan, and/or FDG-PET/CT. Written informed consent was obtained from all patients.

Throughout the study period, simulation and treatment planning techniques evolved in accordance with institutional resources and technological advancements. Until 2017, all patients were treated using conventional three-dimensional computed tomography (3D-CT)-based planning. In this setting, three separate CT scans were acquired: one during deep inspiration, one during deep expiration, and one during free breathing. These datasets were fused to construct the internal target volume (ITV), accounting for respiratory motion.

From 2017 onwards, advanced respiratory motion management strategies were introduced into routine clinical practice. In medically fit and cooperative patients, Deep Inspiration Breath Hold (DIBH) was the preferred method to minimize tumor motion and reduce radiation exposure to adjacent healthy tissues. When DIBH was not feasible, four-dimensional CT (4D-CT) was utilized to evaluate respiratory-induced tumor motion. If neither DIBH nor 4D-CT could be implemented, the conventional 3D-CT planning technique, as previously described, was applied.

Target volume delineation was tailored according to the imaging modality. For patients planned with DIBH, the gross tumor volume (GTV) was first delineated, followed by a 5–7 mm expansion to define the clinical target volume (CTV). Notably, anatomical structures without tumor involvement—such as bone, esophagus, or heart—were excluded from the CTV, even if within the expansion margin. An additional 5–7 mm margin was then applied to generate the planning target volume (PTV). For 4D-CT-based planning, maximum intensity projection (MIP) images reconstructed from 10-phase respiratory-correlated scans were used to define the ITV, encompassing the full extent of tumor motion. A uniform 5 mm expansion was applied to create the PTV.

All planning CT datasets were fused with diagnostic FDG-PET/CT scans to ensure accurate target delineation. The prescribed dose was consistently delivered to the 80% isodose line, normalized to the maximum dose. Treatments were administered using a linear accelerator with 6‑MV photon beams, employing 7 to 9 static, non-opposing coplanar fields. Patient positioning was verified prior to each treatment session using in-room kilovoltage cone-beam CT (CBCT), when available.

The heterogeneity observed in single-fraction dose prescriptions can be attributed to the extended inclusion period (2000–2023) and an individualized treatment approach tailored to patient-specific factors. In the early years of the study, there was no universally accepted standard for single-fraction SBRT in NSCLC. Dose selection was influenced by clinical considerations including advanced patient age, compromised pulmonary function, significant comorbidities, central tumor location, tumor volume, and available institutional technology. Additionally, PET/CT findings and multidisciplinary tumor board recommendations played an important role in guiding dosing decisions. In all cases, the 80% isodose line was used for prescription to ensure adequate target coverage while minimizing radiation exposure to surrounding normal tissues.

Following treatment, patients were evaluated for adverse events at each visit using the Common Terminology Criteria for Adverse Events version 4.0 (CTCAE v 4.0). The initial post-treatment assessment was conducted 8–12 weeks after SBRT using a chest CT scan. Thereafter, chest CT scans were performed every 3 months during the first 2 years and every 6 months thereafter.

The definition of survival outcomes was as follows: LC was the time to recurrence within the treated area or adjacent and overlapped with the PTV; PFS was defined as the time to either local or distant progression or death; out-of-field recurrence was defined as recurrence in any area outside the treated lesion. OS was defined as the interval between the date of initial diagnosis—established either histologically or radiologically—and the date of death or the last recorded follow-up, whichever occurred first.

Survival estimates were calculated using the Kaplan-Meier method. Prognostic parameters included age, sex, histology, lesion diameter, PTV size, response type, and severe toxicity. Univariate analysis was performed using either the log-rank test or Cox regression for continuous variables. Clinically relevant variables were included in the multivariate analysis if they showed significance at *p* ≤ 0.2 in univariate analysis. Statistical analyses were conducted using SPSS version 22.0 (SPSS Inc., Chicago, IL), and *p*-values ≤ 0.05 were considered statistically significant.

## Results

### Patient characteristics

A total of 110 patients with 116 pulmonary lesions were included in the analysis. The cohort comprised 76 males and 34 females, with a mean age of 70.1 years (range: 54–87 years). Histological diagnoses were available for 86 lesions (74.1%): 36 (31.0%) adenocarcinomas, 45 (38.8%) squamous cell carcinomas, and 5 (4.3%) large cell carcinomas. The remaining 30 lesions (25.9%) were CT-morphologically defined round lesions treated without histological confirmation.

Histological sampling was not performed in these cases primarily due to patient-related factors such as advanced age, severe comorbidities, or refusal to undergo invasive diagnostic procedures. In all of these patients, the morphological appearance on CT imaging was highly suggestive of malignancy. Additionally, treatment decisions were supported by further clinical and radiological findings such as FDG-PET/CT uptake, documented lesion growth over time, and/or high-risk clinical profiles.

FDG-PET/CT imaging was available in 51 cases, with a mean maximum standardized uptake value (SUVmax) of 6.65 (range: 1–24.2). The PTV ranged from 3.1 to 47.9 cm^3^ (mean: 12.62 cm^3^). Patients were grouped by prescribed SBRT dose: 89 lesions (76.7%) in 87 patients received ≥ 30 Gy, and 27 lesions (23.3%) in 24 patients received < 30 Gy. Table 1 provides a summary of the patient characteristics.

**Table 1:** Overview of patient characteristics (*N* = 110)

Parameter	*n* (%) or Value	Additional Notes
*Total patients/lesions*	110 patients/116 lesions	–
*Gender*	Male: 76 (69%) Female: 34 (31%)	–
*Age*	Mean: 70.1 years Median: 70 years Range: 54–87 years	–
*Histology*	Squamous cell carcinoma: 41 (37.3%) Adenocarcinoma: 36 (32.7%) Large cell carcinoma: 5 (4.5%) CT-morphologically defined lesions without histological confirmation, considered as NSCLC: 28 (25.5%)	Percentages are based on patients
*PET-CT prior to SBRT*	51 patients (46.4%)	Mean SUVmax: 6.65 (range: 1–24.2)
*PTV*	Mean: 12.62 cm3 Range: 3.1–47.9 cm3	–
*Follow-up duration*	Mean: 30 months Median: 20.5 months	–
*Prescribed dose groups*	1 × 30 Gy: 85 lesions (73.3%) 1 × 25 Gy: 10 lesions (8.6%) Dose ≥ 30 Gy: 89 lesions (76.7%) Dose < 30 Gy: 27 lesions (23.3%)	Range: 18–33 Gy Mean: 28.3 Gy Median: 30 Gy
*Pneumonitis*	Acute (≤ 6 weeks): 2 patients (1.8%) Late (> 6 weeks): 22 patients (20%)	All grade 1–2
*Post-treatment mortality*	36 patients (32.7%)	–

NSCLC Non-Small Cell Lung Cancer, PET-CT Positron Emission Tomography—Computed Tomography, SBRT Single-Fraction Stereotactic Body Radiotherapy, PTV Planning Target Volume

### Toxicity

Treatment-related toxicity was minimal. Acute pneumonitis (within 6 weeks post-SBRT) was observed in only 2 patients (1.8%). During longer follow-up, an additional 22 patients developed pneumonitis, resulting in a total incidence of 21%. All of these cases occurred beyond the acute phase and were considered late-onset pneumonitis. All toxicities were classified as CTCAE grade 1–2. Management was based on symptom severity: some patients with mild symptoms were managed by close observation alone, while others received corticosteroids and/or antibiotic therapy. No grade ≥ 3 toxicities were observed.

When stratified by planning technique, 71 patients with 77 lesions treated before 2017 received 3D-CT-based planning. In this subgroup, 2 patients (2.8%) developed acute pneumonitis and 17 patients (23.9%) developed late-onset pneumonitis. Among the 39 patients treated after 2017—primarily using advanced motion management techniques such as DIBH or 4D-CT—only 5 patients (12.8%) developed radiation-induced pneumonitis. Notably, only 3 of these patients received 25 Gy, while the remaining 36 were treated with a single 30 Gy fraction.

It is important to emphasize that only radiologically confirmed inflammatory changes were classified as pneumonitis. Imaging findings consistent with fibrosis or scar formation, in the absence of associated inflammatory features, were not included in this category.

### Oncological outcomes

The mean follow-up (FU) duration for the overall cohort was 30 months (median: 20.5 months). At the time of data cutoff, 36 patients (32.7%) had died. Local failure (in-field) occurred in 21 patients with 22 lesions (10.0%), while 43 patients with 45 lesions (38.8%) experienced out-of-field progression. Patterns of progression included pulmonary recurrence in 25 patients (22.7%), lymph node relapse in 7 patients (6.4%), brain metastases in 4 patients (3.6%), liver metastases in 2 patients (1.8%), bone metastasis in 1 patient (0.9%), and multiple-site progression in 7 patients (6.4%).

In the high-dose group (≥ 30 Gy), the mean FU was 30.03 months (median: 19 months). At data cutoff, 27 out of 87 patients (31.0%) had died. Local failure was observed in 13 patients (13 lesions, 10.2%), while out-of-field progression occurred in 30 patients (31 lesions, 34.8%).

In the low-dose group (< 30 Gy), the mean FU was also 30.03 months. At data cutoff, 9 out of 24 patients (37.5%) had died. Local failure occurred in 8 patients (9 lesions, 33.3%), and out-of-field progression was observed in 12 patients (14 lesions, 51.9%).

### Survival outcomes

**Fig. 1:**
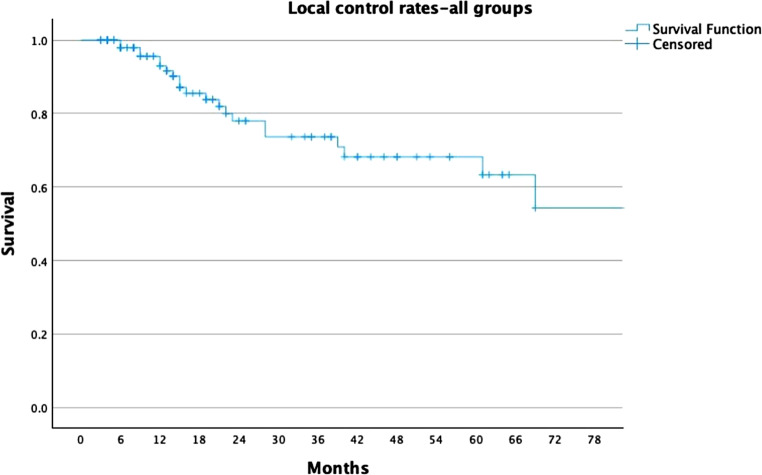
Local Control—All Patients Kaplan-Meier curve showing local control rates for the entire cohort

**Fig. 2:**
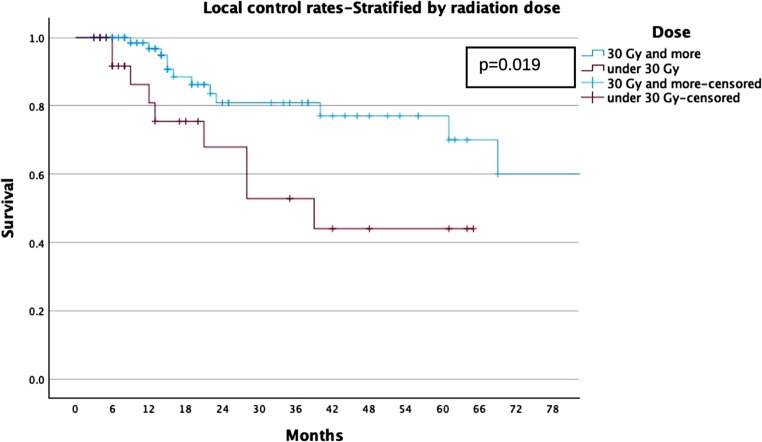
Local Control—Stratified by Radiation Dose Kaplan-Meier curve comparing local control between patients receiving ≥ 30 Gy vs. < 30 Gy

**Fig. 3:**
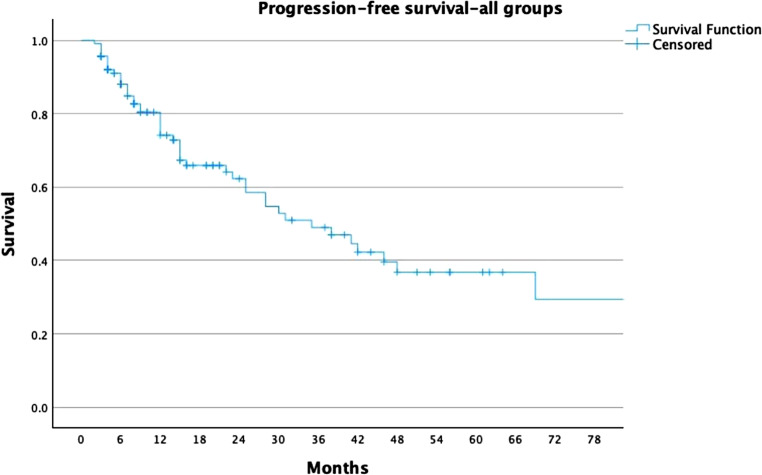
Progression-Free Survival—All Patients Kaplan-Meier curve showing progression-free survival for all patients

**Fig. 4:**
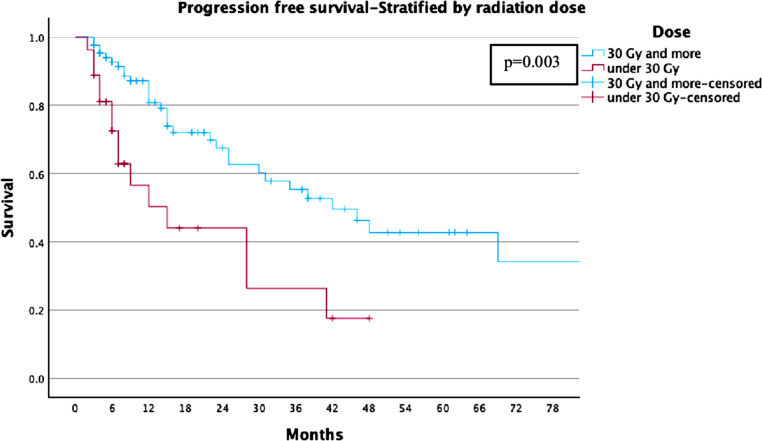
Progression-Free Survival—Stratified by Radiation Dose Kaplan-Meier curve comparing PFS between ≥ 30 Gy and < 30 Gy groups

**Fig. 5:**
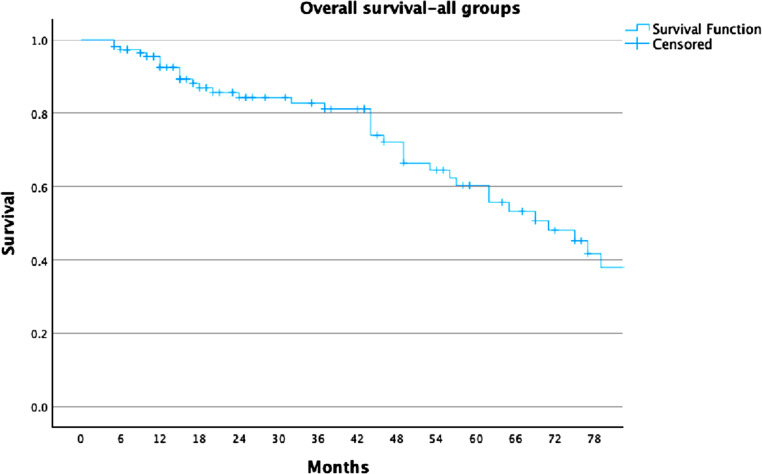
Overall Survival—All Patients Kaplan-Meier curve showing overall survival for the entire cohort

**Fig. 6:**
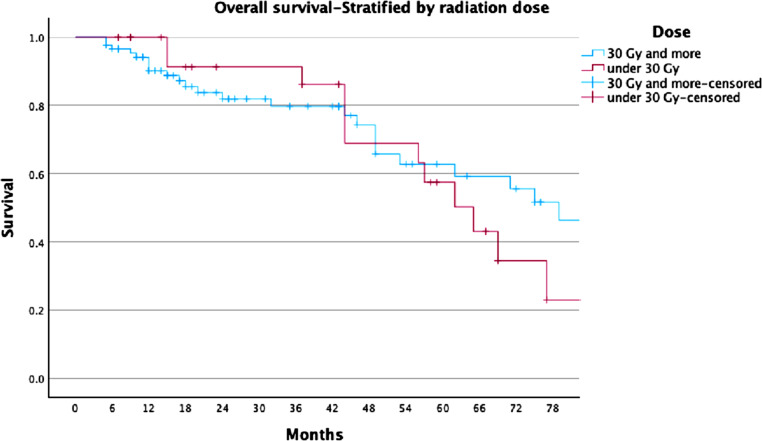
Overall Survival—Stratified by Radiation Dose Kaplan-Meier curve comparing OS between patients treated with ≥ 30 Gy vs. < 30 Gy

For the whole cohort, LC rates were 78%, 74%, and 68% at 2, 3, and 5 years, respectively (Fig. 1). For the ≥ 30 Gy group, LC rates were 81%, 81%, and 77%, compared to 68%, 53%, and 44% in the < 30 Gy group (Fig. 2). PFS rates for the entire cohort were 63%, 49%, and 37% at 2, 3, and 5 years, respectively (Fig. 3). In the ≥ 30 Gy group, PFS rates were 68%, 56%, and 43%, compared to 43%, 26%, and 17% in the < 30 Gy group (Fig. 4). The whole cohort’s overall survival (OS) rates were 84%, 83%, and 60% at 2, 3, and 5 years, respectively (Fig. 5). For the ≥ 30 Gy group, OS rates were 84%, 80%, and 63%, while in the < 30 Gy group, rates were 91%, 83%, and 57% (Fig. 6).

Table 2 presents a summary of the outcomes for local control, progression-free survival, and overall survival.

**Table 2:** Overview of results of local control, progression-free survival and overall survival

Local control		All	≥ 30 Gy	< 30 Gy
–	At 2 years	78	81	68
–	At 3 years	74	81	53
–	At 5 years	68	77	44
*Progression-Free Survival*				
–	At 2 years	63	68	43
–	At 3 years	49	56	26
–	At 5 years	37	43	17
*Overall Survival*				
–	At 2 years	84	84	91
–	At 3 years	83	80	83
–	At 5 years	60	63	57

### Influence of comorbidities and dose

**Fig. 7:**
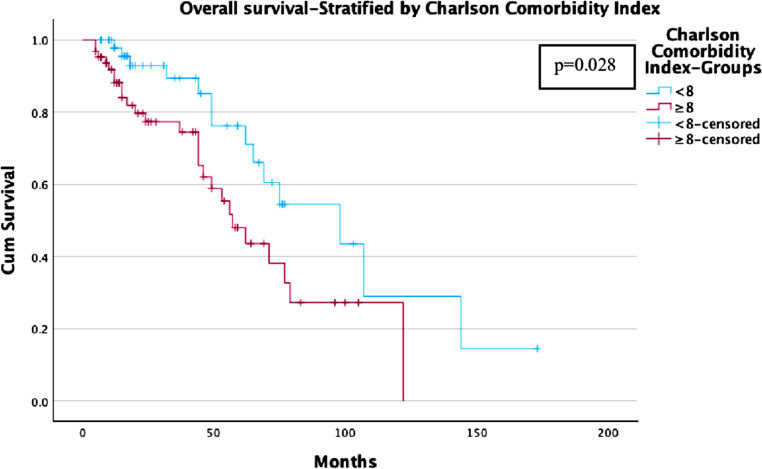
Overall Survival—Stratified by Charlson Comorbidity Index Kaplan-Meier curve comparing OS between patients with Charlson Comorbidity Index < 8 vs. ≥ 8

Patients with lower Charlson Comorbidity Index (CCI < 8) showed better survival rates compared to those with higher comorbidity burden (CCI ≥ 8). In the ≥ 30 Gy group, the OS was 90% at 2 years, 85% at 3 years, and 70% at 5 years in the low CCI (< 8) group. While, the OS was 60% at 2 years, 50% at 3 years, and 35% at 5 years in patients with high CCI (≥ 8) (Fig. 7).

Cox proportional hazards regression models were conducted using a forward stepwise likelihood ratio method, to evaluate the influence of Age at Radiotherapy (RT), the CCI, the dose per fraction (< 30 Gy vs. ≥ 30 Gy) and the sex predictors of LC, PFS and OS. For both LC and PFS, the dose per fraction emerged as significant predictor of tumor control with a dose ≥ 30 Gy leading to improved LC (Exp (B) = 0.349, *p* = 0.019, 95% CI [0.145, 0.843]) and PFS (Exp (B) = 0.382, *p* = 0.003, 95% CI [0.204, 0.715]) while sex, age and the CCI had no significant influence in this model. Regarding the OS, no significant influence of the SBRT-dose was found, neither had the sex a significant influence on the OS-Status. However, increasing age (Exp (B) = 1.101, *p* < 0.001, 95% CI [1.045, 1.161]) and severity of comorbidities (Exp (B) = 1.178, *p* < 0.03, 95% CI [1.014, 1.368]) proved to significantly predict lower OS.

Histopathological confirmation did not have a statistically significant impact on clinical outcomes, including overall survival (OS) (HR = 0.847; 95% CI: 0.429–1.673; *p* = 0.632), progression-free survival (PFS) (HR = 1.240; 95% CI: 0.614–2.504; *p* = 0.549), or local control (LC) (HR = 1.166; 95% CI: 0.426–3.197; *p* = 0.765).

Table 3 presents the results of the Cox regression analysis identifying predictors of local control, progression-free survival, and overall survival.

**Table 3:** Cox regression analysis of predictors of local control, progression-free survival and overall survival

*LC*		
	*HR, CI*	*p‑value*
Dose per fraction: ≥ 30 Gy (*n* = 89) vs. < 30 Gy (*n* = 27)	0.35, 0.15–0.84	0.019
Sex: Male (*n* = 76) vs. female (*n* = 34)		0.24
Age at SBRT		0.49
Charlson Comorbidity index: ≥ 8 (*n* = 61) vs. < 8 (*n* = 49)		0.62
*PFS*		
Dose per fraction: ≥ 30 Gy (*n* = 89) vs. < 30 Gy (*n* = 27)	0.38, 0.2–0.72	0.003
Sex: Male (*n* = 76) vs. female (*n* = 34)		0.69
Age at SBRT		0.66
Charlson Comorbidity index: ≥ 8 (*n* = 61) vs. < 8 (*n* = 49)		0.59
*OS*		
Dose per fraction: ≥ 30 Gy (*n* = 89) vs. < 30 Gy (*n* = 27)	–	0.99
Sex. Male (*n* = 76) vs. female (*n* = 34)	–	0.32
Age at SBRT	1.1, 1.05–1.16	< 0.001
Charlson Comorbidity index ≥ 8 (*n* = 61) vs. < 8 (*n* = 49)	1.2, 1.01–1.37	0.028

HR Hazard ratio, CI confidence interval, *p* = *p*-value. HR reported for *p* < 0.05, LC local control, PFS progression free survival, OS overall survival, SBRT Single-Fraction Stereotactic Body Radiotherapy, HR Hazard ratio, CI confidence interval

*p*: *p*-value

HR reported for *p* < 0.05

## Discussion

This study highlights the efficacy and safety of single-fraction SBRT (SF-SBRT) for primary NSCLC, with a specific focus on doses ≥ 30 Gy. Our findings demonstrate robust long-term LC and PFS, along with minimal toxicity, underscoring SF-SBRT as a viable alternative to multi-fraction protocols for select patient populations. The LC rates of 81% and 77% at 3, and 5 years for patients treated with ≥ 30 Gy affirm the dose-dependent effectiveness of SF-SBRT. These outcomes are consistent with those reported in previous studies, such as RTOG 0915 and others, which documented similar efficacy in dose-escalation trials [12, 13]. By contrast, patients receiving doses < 30 Gy exhibited significantly lower LC rates (53%, and 44% at 3, and 5 years), emphasizing the importance of dose optimization for achieving optimal tumor control. The PFS and OS analyses further support the superiority of higher doses. At 5 years, patients treated with ≥ 30 Gy achieved a PFS of 43%, compared to 17% in the < 30 Gy cohort. These findings corroborate previous research indicating that biologically effective dose (BED) thresholds, such as BED10 ≥ 100 Gy, play a crucial role in enhancing both local and systemic outcomes.

Our findings align closely with those of Singh et al., who reported comparable PFS, and OS rates between single-fraction and multi-fraction SBRT regimens for early-stage NSCLC [13]. Specifically, our 2‑year PFS of 68% for ≥ 30 Gy aligns with Singh’s 65% for a single 30 Gy fraction. However, our 2‑year OS of 84% exceeds their reported 73%, potentially reflecting differences in patient selection or comorbidities. Notably, our study demonstrated lower toxicity, with only 2% acute pneumonitis and no grade ≥ 3 events, compared to 16% thoracic grade 3 adverse events in Singh’s single-fraction cohort. These results reaffirm the efficacy and safety of high-dose single-fraction SBRT and highlight its advantages in reducing treatment burden while maintaining excellent clinical outcomes.

Our results are inferior with those of the RTOG 0915 trial, which compared single-fraction SBRT (34 Gy) to multi-fraction SBRT (48 Gy in 4 fractions) in patients with peripheral NSCLC. Both studies demonstrated high long-term local control rates, with RTOG reporting 5‑year primary tumor failure rates of 10.6% and 6.8% for single- and multi-fraction arms, respectively, comparable to the 77% 5‑year local control achieved in our high-dose single-fraction cohort. PFS and OS also align, with RTOG showing median OS of 4.1–4.6 years and 5‑year OS of 29.6%–41.1%, similar to the 5‑year OS of 63% observed in our high-dose group. Importantly, both studies confirmed the low toxicity profile of single-fraction SBRT, with RTOG reporting 2.6% grade 3 toxicity, comparable to our finding of no grade 3 or higher events. These findings collectively reinforce the efficacy and safety of single-fraction SBRT for early-stage, inoperable NSCLC, offering excellent clinical outcomes with reduced treatment burden.

Stanford University released the initial prospective studies on dose-escalation in 2003. The authors subsequently reported the results at a variety of concentrations [14, 15]. The research design of this Phase I trial was centered on dosage escalation, with four doses of single-fraction ranging from 15 Gy to 30 Gy, gradually increasing by 5 Gy.

The primary goal was to determine the maximum tolerated dose (MTD) of SF three months after administration. There was a total of 32 inoperable patients, 20 of whom had NSCLC and 12 of whom had metastatic lesions that were less than 5 cm in size. Individuals with central tumors and a PTV greater than 50 cc exhibited Grade 2–3 pneumonitis after 5–6 months. In contrast, the administration of 25 Gy to patients who had a history of radiation led to a significant increase in the severity of the adverse effects. In order to exclude individuals with a PTV greater than 50 cc who had previously undergone radiation therapy, an additional modification was made to the 30 Gy dose. The three G5 toxicities that were recorded were linked to centrally located tumors in patients who had previously undergone chemotherapy. One patient received treatment prior to SABR, while the other two received it as adjuvant therapy to SBRT. Additionally, two of the patients had a PTV exceeding 50 cc. At one year, the LC rate was 91% for patients who received a dose exceeding 20 Gy and 54% for those who received a dose below 20 Gy. Metastatic lesions exhibited significantly inferior LC in comparison to basic tumors. The authors have determined that SF SBRT at a dosage of 25 Gy is well-tolerated in patients with a history of thoracic radiotherapy and a PTV of less than 50 cc. However, the cohort that has undergone prior chemotherapy, either prior to or following SBRT, and central lesions may be at an increased risk.

Additionally, this study is the first to assess the Charlson Comorbidity Index (CCI) as a predictor of OS. The Cox proportional hazards analysis identified the Kaplan-Meier method as the sole factor to exhibit a statistically significant trend, demonstrating improved OS for patients with CCI scores less than 8. This suggests that the primary determinant of OS in this relatively frail patient cohort is overall health status. Furthermore, it underscores the utility of the CCI in this context.

A German study has similarly focused on evaluating prognosis in patients with early-stage lung cancer using the Age-adjusted Charlson Comorbidity Index (aCCI) [16]. Dreyer et al. analyzed baseline comorbidities and their impact on clinical outcomes in a cohort of 196 patients. The median OS was 15.0 months, attributed to advanced age and competing comorbidities. The aCCI was used to assess prognosis, revealing that patients with an aCCI of 8 or higher had significantly higher risks of death and cancer-specific mortality than those with an aCCI of 7 or lower. Compared to the Cumulative Illness Rating Scale for Geriatrics (CIRS-G), the aCCI is quicker and more accessible for clinical application. Although the CIRS‑G has not been widely used in large cohorts of medically inoperable lung cancer patients treated with SBRT, the aCCI effectively assessed survival outcomes in this population. It was demonstrated to be a reliable prognostic tool. Furthermore, Dreyer et al. reported that the clinically relevant radiation pneumonitis ≥ 2 rate was 12.7% in this primarily multimorbid patient collective, which was not abnormally elevated. In this context, Kowalchuk et al. identified three tumor-specific factors that warrant further investigation for their potential influence on OS: BED, tumor size, and pre-treatment SUV [17]. Their research revealed a Kaplan-Meier analysis trend and an OS benefit with increased BED (≥ 120 Gy) in univariate analysis. Interestingly, this threshold is higher than that of earlier studies, which observed improved LC with BED10 ≥ 100 Gy [18]. However, in our study, treatments with BED10 ≥ 100 Gy did not demonstrate a corresponding improvement in OS.

These findings align with those of Pennathur et al. [19], who conducted a retrospective study on 100 patients treated with SBRT for recurrent tumors using various regimens, including single-fraction SBRT and multi-fraction schedules (45–60 Gy in 3–5 fractions). They reported 5‑year OS rates of approximately 57%, with 1‑, 2‑, and 5‑year OS rates of 74%, 49%, and 31%, respectively, and a median follow-up of 51 months. This study demonstrated promising outcomes for SBRT in oligo-recurrent or oligo-progressive lung cancer, with no significant adverse effects.

In our cohort, multivariate analysis indicated that comorbidities and age significantly influenced OS, highlighting the importance of patient selection to achieve optimal long-term outcomes. Our study’s safety profile is also noteworthy, with no grade 3 or higher toxicities and a late pneumonitis rate of 21%.

These findings are consistent with those of Tekatli et al. [20], who reported similarly low toxicity rates (≤ 2%) for single-fraction SBRT in patients with synchronous lung malignancies. In their retrospective analysis of 84 patients with 188 pulmonary lesions treated for primary and metastatic synchronous tumors, only seven lesions (3.7%) were treated with a single 34 Gy fraction using multicentric VMAT for spatially separated lesions. Grade 3 or higher toxicities were observed in just 2% of patients. In our study, even with high-dose regimens, safety was evident, with acute pneumonitis occurring in only two cases.

Additionally, Kumar et al. [21] compiled data on 445 patients with early-stage NSCLC treated with SBRT at the Cleveland Clinic. This cohort included 26 patients (5.8%) with synchronous lung tumors confirmed by biopsy and/or PET-CT. All patients, whether treated for synchronous or solitary tumors, received single-fraction SBRT at doses of 30 Gy or 34 Gy. After one year, no significant differences in progression or survival rates were observed between the two groups.

Single-fraction SBRT is also economically advantageous. In light of rising healthcare costs, cost-effective and efficient RT options should be prioritized. This is particularly relevant in the United States, where the Centers for Medicare and Medicaid Services are transitioning from fee-for-service to episode-based payment models to improve cost efficiency [22]. SF SBRT offers substantial financial savings, as its costs are estimated to be 40% lower than those of three-fraction regimens, based on 2009 Medicare prices [23]. During the COVID-19 pandemic, single-fraction regimens can further reduce costs by minimizing the use of personal protective equipment and clinical resources. This study acknowledges its limitations, including its retrospective design, single-institution scope, and lack of comprehensive toxicity data. Additionally, certain NSCLC subtypes, such as lepidic pattern adenocarcinoma, which is characterized by its indolent behavior and lower metastatic potential, were excluded [24, 25]. However, our findings indicate that toxicity associated with single-fraction SBRT, even in chest wall cases, remains low. Future prospective, multicenter trials are needed to validate these results, refine patient selection criteria, and explore biomarkers for treatment response.

While our findings indicate favorable long-term outcomes and a strong safety profile for high-dose single-fraction SBRT in NSCLC, several limitations must be acknowledged. Most importantly, the reliability of LC, PFS, and OS outcomes is highest in patients with histologically confirmed NSCLC. In patients without biopsy confirmation, even FDG-PET-positive lesions may represent benign conditions such as inflammation or infection, which can radiologically mimic malignancy. Consequently, the inclusion of PET-positive but histologically unverified lesions introduces the risk of misclassifying benign nodules as malignant, potentially leading to an overestimation of LC and PFS following SBRT. To account for this limitation, we conducted separate analyses for the entire cohort and for the subset of patients with histologically confirmed diagnoses. Future prospective studies should prioritize histopathologic confirmation to ensure the validity and accuracy of survival outcomes and treatment efficacy assessments.

Table 4 provides a literature review of studies involving non-small-cell lung cancer patients treated with stereotactic radiotherapy.

**Table 4:** Literature review for non-small-cell lung cancer patients treated with stereotactic radiotherapy

Study	Number of patients	*Follow-up* (Median) Months	Age (Median)	Dose (Gy)/Fx	LC (%)	PFS (%)	OS (%)
*Videtic et al., 2019 (RTOG 0915) *[7]	84	48	75	34/1 or 48/4	97–93 at 1‑y; 5‑y primary failure rate, 11–7	Median 2.6 y arm 1, 2.8 y arm 2. 19 arm 1, 33 arm 2 at 5 y	85–91 at 1 y, 61–78 at 2 y. 30–41 at 5 y
*Singh et al., 2019 *[17]	58	54	71	30/1 or 60/3	95–97 at 2 y	65–50 at 2 y	73–62 at 2 y
*Iovoli et al., 2023* [25]	263	27.2	76	27/1–30/1–34/1	92–87.3 at 2 and 5 y	54.7–22 at 2 and 5 y	65.1–25.7 at 2 and 5 y
*Huang et al. 2023* [26]	265	44.2	77	27/1-50/5	5‑y LC: Central 92.9, Peripheral 92.5	23.3–22.3 at 5 y	29.3–28.3 at 5 y
*Nicosia et al., 2019* [27]	44	34	75	30/1	87.8–87.8 at 3 and 5 y	65.5 and 56.7 at 3 and 5 y	64.9 and 36.9 at 3 and 5 y
*Hof et al., 2003 *[28]	10	14.9	71	19–26/1	80 at 14.9 mo.	LRFS 89 at 12 mo., 71.1 at 24 mo.	80 at 1 y and 64 at 2 y
*This Study *[29]	111	20.5	70	15–33/1	≥ 30 Gy: 81, 81, and 77 at 2, 3, and 5 y < 30 Gy 68, 53, and 44 at 2, 3, and 5 y	≥ 30 Gy: 68, 56, and 43 at 2, 3, and 5 y. < 30 Gy 43, 26, and 17 at 2, 3, and 4 y	≥ 30 Gy: 84, 80, and 63 at 2, 3, and 5 y. < 30 Gy 91, 83, and 57 at 2, 3, and 5 y

Fx Number of fractionsLC Local contro, PFS progression-free surviva, OS Overall surviva, y year

## Conclusion

High-dose (≥ 30 Gy) single-fraction stereotactic body radiotherapy (SF-SBRT) offers an effective, well-tolerated, and logistically convenient treatment option for patients with early-stage NSCLC, particularly those who are medically inoperable due to age or comorbidities. This study demonstrated excellent long-term local control and progression-free survival with minimal toxicity. Outcomes in the ≥ 30 Gy group were significantly superior in terms of LC, PFS, and OS compared to lower-dose treatment. Moreover, the use of modern motion management techniques such as DIBH and 4D-CT was associated with a lower incidence of radiation-induced pneumonitis than conventional 3D-CT planning. Importantly, the analysis highlighted the prognostic significance of comorbidities, with higher Charlson Comorbidity Index (CCI) scores correlating with reduced overall survival, irrespective of SBRT dose. These findings support the broader adoption of high-dose SF-SBRT in appropriately selected patients and underscore the importance of individualized treatment planning. Prospective trials are warranted to validate these results and refine selection criteria further.
