# Correction to: From protocol to practice: long-Term outcomes of single-Fraction stereotactic body radiotherapy for primary non-Small cell lung cancer

**DOI:** 10.1007/s00066-025-02485-x

**Published:** 2025-11-19

**Authors:** Kerem Tuna Tas, Philipp Lishewski, Fatima Frosan Sheikhzadeh, Edgar Smalec, Niklas Recknagel, Thomas Wündisch, Angelique Holland, Andreas Kirschbaum, Khaled Elsayad, Rita Engenhart-Cabillic, Klemens Zink, Hilke Vorwerk, Sebastian Adeberg, Ahmed Gawish

**Affiliations:** 1 Department of Radiotherapy and Radiation Oncology, Marburg University Hospital, Marburg, Germany; 2 Department of Radiotherapy and Radiation Oncology, Philipps-Universität Marburg, Marburg, Germany; 3 Marburg Ion-Beam Therapy Center (MIT), Department of Radiotherapy and Radiation Oncology, Marburg University Hospital, Marburg, Germany; 4 University Cancer Center (UCT) Frankfurt—Marburg, Marburg, Germany; 5 LOEWE Research Cluster for Advanced Medical Physics in Imaging and Therapy, (ADMIT), TH Mittel Hessen University of Applied Sciences, Giessen, Germany; 6 Department of Medicine, Pulmonary and Critical Care Medicine, University Medical Center Giessen and Marburg, Philipps-University Marburg, Marburg, Germany; 7 Department of Visceral, Thoracic and Vascular Surgery, University Hospital Gießen and Marburg (UKGM), Marburg, Germany

**Correction to:**

**Strahlenther Onkol 2025**

10.1007/s00066-025-02462-4

In this article, the author’s name Khaled Elsayad was incorrectly written as Khalid Elsayad, and the author’s name Philipp Lishewski was incorrectly written as Phillip Lishewski.

The original article has been corrected.

